# Combined approaches for increasing fetal hemoglobin (HbF) and *de novo* production of adult hemoglobin (HbA) in erythroid cells from β-thalassemia patients: treatment with HbF inducers and CRISPR-Cas9 based genome editing

**DOI:** 10.3389/fgeed.2023.1204536

**Published:** 2023-07-17

**Authors:** Alessia Finotti, Roberto Gambari

**Affiliations:** ^1^ Center “Chiara Gemmo and Elio Zago” for the Research on Thalassemia, University of Ferrara, Ferrara, Italy; ^2^ Department of Life Sciences and Biotechnology, University of Ferrara, Ferrara, Italy

**Keywords:** β-thalassemia, gene editing, CRISPR-Cas9, fetal hemoglobin (HbF), adult hemoglobin

## Abstract

Genome editing (GE) is one of the most efficient and useful molecular approaches to correct the effects of gene mutations in hereditary monogenetic diseases, including β-thalassemia. CRISPR-Cas9 gene editing has been proposed for effective correction of the β-thalassemia mutation, obtaining high-level “*de novo*” production of adult hemoglobin (HbA). In addition to the correction of the primary gene mutations causing β-thalassemia, several reports demonstrate that gene editing can be employed to increase fetal hemoglobin (HbF), obtaining important clinical benefits in treated β-thalassemia patients. This important objective can be achieved through CRISPR-Cas9 disruption of genes encoding transcriptional repressors of γ-globin gene expression (such as *BCL11A, SOX6, KLF-1*) or their binding sites in the HBG promoter, mimicking non-deletional and deletional HPFH mutations. These two approaches (β-globin gene correction and genome editing of the genes encoding repressors of γ-globin gene transcription) can be, at least in theory, combined. However, since multiplex CRISPR-Cas9 gene editing is associated with documented evidence concerning possible genotoxicity, this review is focused on the possibility to combine pharmacologically-mediated HbF induction protocols with the “*de novo*” production of HbA using CRISPR-Cas9 gene editing.

## 1 Introduction

The β-thalassemias are a genetically heterogenous group of hereditary hematological diseases caused by hundreds of mutations of the adult β-globin gene, leading to low or absent production of adult hemoglobin (HbA) in erythroid cells (Weatherall, 2001; Galanello and Origa, 2010; Fucharoen and Weatherall, 2016; Origa, 2017). The thalassemia syndromes, together with sickle-cell disease (SCD), are impactful diseases especially in developing countries, where they maintain a very high frequency within the population, due to the lack of genetic counselling and prenatal diagnosis (Weatherall, 2001; Origa, 2017). Regular blood transfusion, chelation therapy and bone marrow transplantation (Fucharoen and Weatherall, 2016) are currently employed for the clinical management of β-thalassemia patients. Alternatively, induction of fetal hemoglobin (HbF) can be considered (Forget, 1998; Musallam et al., 2012; Liu et al., 2014; Sripichai and Fucharoen, 2016a), taking into account the increasing number of laboratory and clinical evidences demonstrating that reactivation of HbF production in adult life can be beneficial for β-thalassemia, leading in some cases to transfusion-independency (Sripichai and Fucharoen, 2016a). As for other human pathologies and rare diseases, most of the new innovative approaches for developing protocols of possible interest for future treatments of β-thalassemias are focusing on personalized treatments on one hand, and precise targeting on the other. In order to reach these objectives, an exciting strategy recently proposed for β-thalassemia (and other genetic diseases) is genome editing of human hematopoietic stem and progenitor cells (HSPC) (Boulad et al., 2018; Magrin et al., 2019; Ernst et al., 2020; Ali et al., 2021; Ferrari et al., 2021; Karamperis et al., 2021; Rosanwo and Bauer, 2021; Quintana-Bustamante et al., 2022; Eckrich and Frangoul, 2023; Khiabani et al., 2023). In this respect, the Clustered Regularly Interspaced Palindromic Repeats (CRISPR)-Cas9 nuclease system should be considered among the most studied gene editing strategies (Dever et al., 2016; Hu, 2016; Lau, 2018; Papasavva et al., 2019).

In addition to possible applications in the therapeutic field, gene editing and the most recent base editing and prime editing approaches, are powerful tools to identify novel druggable targets. For instance, Ravi NS et al. reported an example describing how CRISPR based editing can be applied to mapping gene regulatory elements in highly homologous loci (in this case the γ-globin gene promoter) (Ravi et al., 2022), strongly supporting the concept that this approach will be in the future a prominent therapeutic strategy for monogenetic disorders, such as β-thalassemia, as demonstrated by the recently published clinical study performed by Frangoul et al., who reported the results on two patients (one with transfusion-dependent thalassemia, the other with sickle-cell disease) who received autologous CD34^+^ cells edited with CRISPR-Cas9 targeting the BCL11A enhancer (ClinicalTrials.gov numbers, NCT03655678 and NCT03745287) (Frangoul et al., 2021). Interestingly, after 1 year follow-up, the β-thalassemia patient exhibited pancellular increases in HbF production and transfusion independence, while in the SCD patient elimination of vaso-occlusive episodes was found (Frangoul et al., 2021).

**FIGURE 1:**
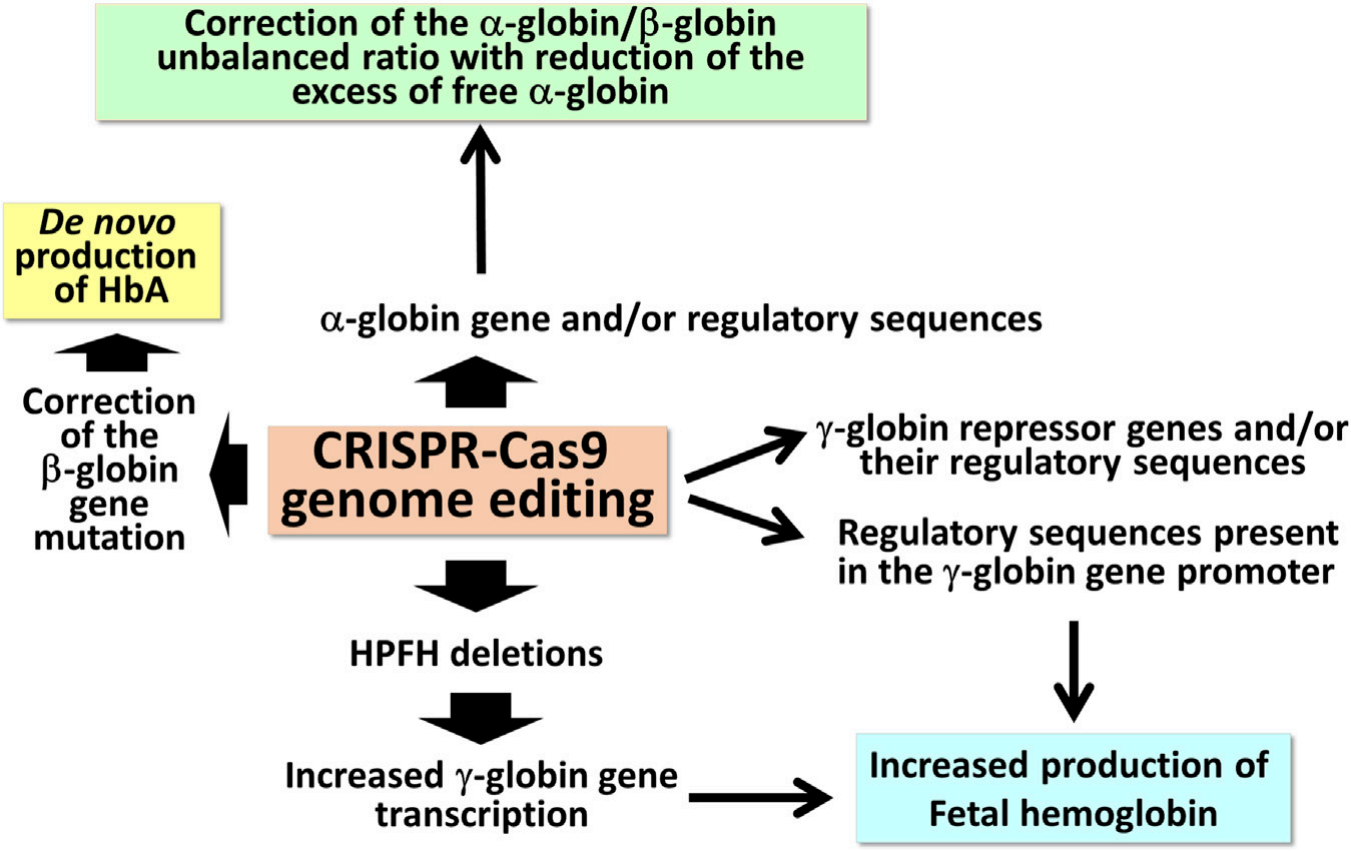
Possible applications of CRISPR-Cas9 based gene editing to achieve end-points of interest in the management of β-thalassemia patients.

The extremely high variety of CRISPR-Cas9 based tailored approaches (Figure 1) opens new avenues in the management of β-thalassemias, also based on combined treatments with the aim to rescue HbA production and, in parallel, reactivate the expression of γ-globin genes, leading to HbF production.

In this short review, we will focus on gene editing based on the CRISPR-Cas9 technology (for which a large number of studies are available in the literature), trying to discuss whether this approach can be suitable for multiple interventions, such as combined treatments to reach “*de novo*” production of HbA together with increased HbF content.

## 2 CRISPR-Cas9 based approaches to reactivate γ-globin gene expression

**FIGURE 2:**
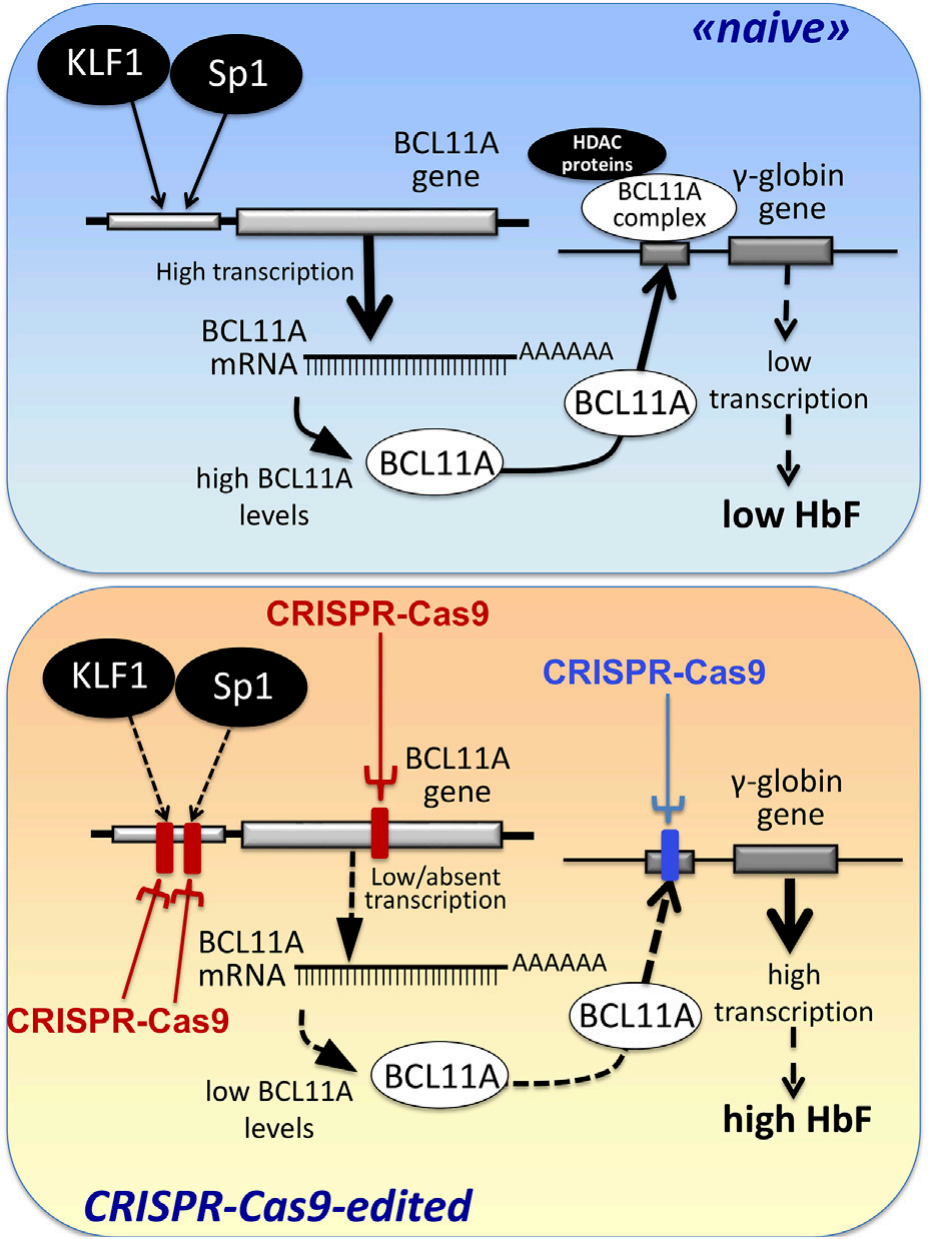
Alternative CRISPR-Cas9 based protocols for the reactivation of the expression of γ-globin genes and the increased production of HbF.

The first observations strongly suggesting that reactivation of the silent γ-globin genes in adult β-thalassemic patients might be highly beneficial to the patients, ameliorating their clinical phenotype, were reported in studies focusing on rare forms of β^0^-thalassemia, associated with large genomic deletions causing HPFH (hereditary persistence of fetal hemoglobin) and identified as “HPFH deletions” in Figure 1; these patients are characterized by absence of β-globin production, but presence of high levels of γ-globin chains, resulting in high levels of HbF associated with a relatively benign clinical course (Forget, 1998; Sharma et al., 2020). More recent clinical studies support the concept that naturally higher production of HbF improves the clinical phenotype of a variety of β-thalassemia patients (Uda et al., 2008; Galanello et al., 2009; Nuinoon et al., 2010; Badens et al., 2011; Danjou et al., 2012). Accordingly, these observations have prompted the activity of a large number of research groups in performing studies on inducers of HbF that can be proposed in clinical trials, as they reproduce to some extent what occurs in β-thalassemia patients with a natural persistence of higher levels of HbF (Gambari and Fibach, 2007; Finotti and Gambari, 2014; Lavelle et al., 2018; Nuamsee et al., 2021). In addition to HbF inducers, reactivation of HbF production can be obtained using different CRISPR-Cas9-based gene editing approaches (Figure 2).

One very interesting strategy has been proposed by several groups trying to reproduce genetic alterations leading to an HPFH in phenotype. For instance, Ye et al. (2016) proposed genome editing using CRISPR-Cas9 to create an HPFH genotype in hematopoietic stem/progenitor cells (HSPCs), obtaining an increase of expression of γ-globin genes. In fact, these gene edited regions were known (or were hypothesized) to contain putative fetal hemoglobin (HbF) silencers, as also pro-posed by Antoniani et al. (2018) and more recently by Venkatesan et al. (2023a).

In the context of gene deletions for increasing expression of γ-globin genes, an interesting study was published by Topfer et al. (2022), who found that CRISPR-Cas9 disruption of the adult β-globin (*HBB*) gene promoter was associated with reactivation of γ-globin gene expression; in these experimental conditions the γ-globin gene outcompetes the *HBB* gene for binding to the LCR (Topfer et al., 2022). The experimental gene editing strategy designed by Topfer and collaborators was based on the observation that in all the deletion-associated HPFH conditions the proximal adult β-globin (HBB) promoter was deleted (Topfer et al., 2022). This study is an excellent validation of the recently proposed model of the switch from fetal γ-globin gene expression to transcription of the adults β-globin gene (Deng et al., 2014). This model is based on the hypothesis that both HBB and HBG promoters compete for the LCR (Carter et al., 2002a; Okamura et al., 2009; Deng et al., 2014). The regulatory mechanisms operating in this model are fundamental for tissue-specific transcriptional control of globin gene expression. The interplay between local DNA regulatory elements and remodeling of chromatin and transcription at the globin gene cluster have been extensively discussed (Carter et al., 2002b; Chakalova et al., 2005) focusing also on the requirement of LCR to open the chromatin. In this context, the disruption of the adult β-globin (HBB) gene promoter leads to deep changes in promoter interactions with the LCR enhancer.

An alternative view has been followed by several studies based on the established concept that γ-globin genes are under the control of transcriptional repressors. The objective, in this case, is a CRISPR-Cas9 based gene editing finalized at a) the knock-out of repressors of γ-globin gene transcription (such as BCL11A) or b) the disruption of their binding sites present within regulatory regions of the β-like gene cluster (including those present within the γ-globin gene). A pictorial representation of these strategies is shown in Figure 2, while a summary of interesting studies in this research field is reported in Table 1 (Canver et al., 2015; Shariati et al., 2016; Martyn et al., 2018; Psatha et al., 2018; Shariati et al., 2018; Khosravi et al., 2019; Weber et al., 2020; Fu et al., 2022; Han et al., 2022; Wakabayashi et al., 2022).

**TABLE 1:** CRISPR-Cas9 gene editing for Fetal Hemoglobin (HbF) reactivation.

Target genomic sequences	Comments	References
Genomic region(s) responsible for the HPFH phenotype	The erythroid colonies differentiated from HSPCs with HPFH deletion showed significantly higher γ-globin gene expression	Ye et al. (2016)
A putative γ-δ intergenic fetal hemoglobin (HbF) silencer	In primary SCD patient-derived hematopoietic stem/progenitor cells, targeting the identified 13.6-kb region results in a high proportion of γ-globin expression in erythroblasts, increased HbF synthesis, and amelioration of the sickling cell phenotype	Antoniani et al. (2018)
A 11-kb sequence, encompassing putative repressor region (PRR) to β-globin exon-1 (βE1)	PRR-βE1 gene editing of patient HSPCs could lead to improved therapeutic outcomes for β-hemoglobinopathy gene therapy	Venkatesan et al. (2023a)
KLF1	Genetic disruption of the KLF1 gene to overexpress the γ-globin gene using the CRISPR/Cas9 system	Shariati et al. (2016)
SOX6 gene	Disruption of SOX6 gene using CRISPR/Cas9 technology for γ-globin reactivation: An approach towards gene therapy of β-thalassemia	Shariati et al. (2018)
BCL11A	Targeted deletion of BCL11A gene by CRISPR-Cas9 system for fetal hemoglobin reactivation: A promising approach for gene therapy of beta thalassemia disease	Khosravi et al. (2019)
BCL11A	CRISPR-Cas9- gene editing of the BCL11A enhancer for pediatric β^0^/β^0^ transfusion-dependent β-thalassemia	Fu et al. (2022)
BCL11A enhancer	BCL11A enhancer dissection by Cas9-mediated *in situ* saturating mutagenesis: identification of regulatory elements	Canver et al. (2015)
BCL11A enhancer	Disruption of the BCL11A Erythroid Enhancer reactivates fetal hemoglobin	Psatha et al. (2018)
BCL11A	CRISPR/Cas9-based multiplex genome editing of BCL11A and HBG efficiently induces fetal hemoglobin expression	Han et al. (2022)
RBM12	Identification and characterization of RBM12 as a novel regulator of fetal hemoglobin expression	Wakabayashi et al. (2022)
HBG gamma-globin promoters	Editing a γ-globin repressor binding site restores fetal hemoglobin synthesis and corrects the sickle-cell disease phenotype	Weber et al. (2020)
Regulatory HBG sequences	Natural regulatory mutations elevate the fetal globin gene via disruption of BCL11A or ZBTB7A binding	Martyn et al. (2018)

### 2.1 Deletion of repressors of γ-globin genes or their enhancer regions

A key study in this field of investigation was reported by Canver et al. (2015), who performed a BCL11A enhancer dissection by Cas9-mediated *in situ* saturating mutagenesis. They used the HUDEP-2 cell line as screening system and primary human CD34^+^ hematopoietic stem and progenitor cells (HSPCs) for validation of the results obtained (Canver et al., 2015). This important study generated a detailed enhancer map, very useful to design therapeutic genome editing strategies. These results were confirmed using another experimental model system by Khosravi et al., who supported the proof-of-principle that deletion(s) of part of the BCL11A gene (including relevant regulatory regions present within the BCL11A enhancer) is (are) associated with the reactivation of HbF production (Khosravi et al., 2019). Using the CRISPR-Cas9 genome-editing strategy on human K562 cells, they deleted a 200 bp genomic region within the *BCL11A* enhancer, obtaining a strong induction of γ-hemoglobin expression.

Along with the same approach, the association between disruption of γ-globin transcriptional repressor genes by gene editing, and reactivation of expression of the γ-globin genes and production of HbF is sustained also by the study performed by Psatha et al. (2018) on BCL11A and by other research groups using CRISPR-Cas9 gene editing on other repressor genes, such as KLF-1 (Shariati et al., 2016), SOX6 (Shariati et al., 2018) and RBM12 (Wakabayashi et al., 2022).

Fu et al. (2022) applied the study to a CRISPR-Cas9-mediated gene editing protocol designed to alter the *BCL11A* erythroid enhancer by disrupting the GATA1-binding site at position +58. Preliminary results of an ongoing phase 1/2 trial (NCT04211480) have been presented in this report, evaluating safety and efficacy of this gene editing therapeutic approach in children with blood transfusion-dependent β-thalassemia (TDT). Concerning safety, when *BCL11A* enhancer-edited, autologous, hematopoietic stem and progenitor cells were transplanted, engraftment was obtained with high efficiency, and adverse events (AEs) were negligible, considered unrelated to gene editing and resolved after specific treatments (Fu et al., 2022). Concerning efficacy, transfusion independency was reached for >18 months after treatment, and Hb increased (Fu et al., 2022). The study by Fu et al. is strongly in agreement with the results obtained by the clinical trials NCT03655678 and NCT03745287 and reported by Frangoul et al. (2021).

Overall, these studies support the concept that disruption of genes encoding a transcriptional repressor of γ-globin genes and/or disruption of their enhancers can be applied and translated into clinical protocols aimed at reactivation of HbF production.

### 2.2 Mutagenesis of transcriptional repressor binding sites of the γ-globin gene promoter: reconstitution of the HPFH phenotype using CRISPR-Cas9 based approaches

Martyn et al. (2018) reported a key study focusing on the binding sites of the γ-globin gene repressors BCL11A and ZBTB7A (also known as LRF), located at −115 and −200 bp from the start of transcription of γ-globin genes. These sites are bound directly by the BCL11A and ZBTB7A repressors. The results obtained demonstrated that the CRISPR-Cas9 based disruption of the repressor binding raised γ-globin gene expression in erythroid cells (Martyn et al., 2018).

## 3 Gene editing for precise correction of the β-globin gene mutations

Table 2 summarizes published gene editing studies aiming at demonstrating highly efficient corrections of the mutated β-globin gene in β-thalassemias (Xu et al., 2015; Liu et al., 2017; Gabr et al., 2020; Cosenza et al., 2021; Lu et al., 2022; Trakarnsanga et al., 2022). The target cells (or cell lines) have been several and heterogenous, confirming the efficacy and reproducibility of the gene editing approach and protocols. These include primary erythroid progenitors isolated from β-thalassemia patients, induced pluripotent stem cells, established, patient-derived cell lines and erythroid cells from β-thalassemia mice carrying selective β-thalassemia mutations. For instance, Trakarnsanga et al. (2022) reported the genetic correction of hemoglobin E in an immortalized hemoglobin E/β-thalassemia cell line using the CRISPR/Cas9 system. This study demonstrated that the HbE-corrected clones restored β-globin production with reduced levels of HbE upon erythroid differentiation. Patient-derived pluripotent stem cells were employed by Liu et al. (2017) to correct a β41-42 (TCTT) deletion mutation. Thalassemic mice were employed by Lu et al. (2022) to demonstrate *in vivo* effects of the correction of the defect of RNA splicing in β654-thalassemia mice using CRISPR/Cas9 gene editing technology. Interestingly, hematologic parameters of all of the edited β654 founders and their offspring were found to be significantly improved compared to those of the control non-edited mice, consistent with the restoration of wild-type β-globin RNA expression (Lu et al., 2022). As a final example, Cosenza et al. reported a CRISPR based approach to obtain efficient correction of the β^0^39-thalassemia mutation in erythroid cells isolated from homozygous β^0^39-thalassemia patients (Cosenza et al., 2021).

**TABLE 2:** Gene editing procedures for HBB mutations.

Gene editing protocol	β-thalassemia target mutation	Comments	References
CRISPR/Cas9 based gene editing	HBB IVS2-654 (C > T)	The HBB IVS2-654 (C > T) mutation was targeted in iPSCs derived from β-thalassemia patients	Xu et al. (2015)
	Hemoglobin E	Genetic correction of haemoglobin E in an immortalised haemoglobin E/beta-thalassaemia cell line using the CRISPR/Cas9 system	Trakarnsanga et al. (2022)
	Splicing defect of β654-thalassemia	Correction of RNA splicing defect in β654-thalassemia mice using CRISPR/Cas9 gene-editing technology	Lu et al. (2022)
	Stop codon β^0^39-thalassemia	Efficient CRISPR-Cas9-based genome editing of β-globin gene on erythroid cells from homozygous β^0^39-thalassemia patients	Cosenza et al. (2021)
	IVS-1-110	CRISPR-mediated gene modification of hematopoietic stem cells with beta-thalassemia IVS-1-110 mutation	Gabr et al. (2020)
	β-41/42 (TCTT) deletion	A study providing an efficient and safe approach for the genetic correction of the β-41/42 (TCTT) deletion in iPSCs derived from β-thalassemia patients	Liu et al. (2017)
Prime editing; base editing	HBB -28 (A>G)	Correction of β-thalassemia mutant by base editor in human embryo	Liang et al. (2017)
	Hemoglobin E	Direct correction of haemoglobin E β-thalassaemia using base editors	Badat et al. (2023)
	IVS-II-654	Prime editing employed	Zhang et al. (2022)
	IVS1-110 (G>A)	Adenine base editor-mediated correction	Hardouin et al. (2023)

In Table 2 a few examples are also reported discussing the newly developed prime and base editing of β-thalassemia mutations (Liang et al., 2017; Zhang et al., 2022; Badat et al., 2023; Hardouin et al., 2023). These novel approaches are expected to limit genotoxic of the gene editing procedures, especially those due to homology-directed repair (HDR), activated following the introduction of DNA double-strand breaks (DSB) during the conventional CRISPR approach (Liang et al., 2017; Komor et al., 2018; Zeng et al., 2020; Collantes et al., 2021; Zhang et al., 2022; Badat et al., 2023; Carusillo et al., 2023; Hardouin et al., 2023).

In respect to the limitations of the CRISPR-Cas9 based procedures, HDR is inherently genotoxic in somatic cells; therefore, the recent development of base editing procedures that edit a target base without requiring the generation of DSB or HDR offers an alternative and very appealing approach (Liang et al., 2017; Zhang et al., 2022; Badat et al., 2023; Hardouin et al., 2023). For instance, Hardouin et al. developed a strategy to correct one of the most prevalent BT mutations (IVS1-110 [G>A]) using the SpRY-ABE8e base editor (Hardouin et al., 2023). These and similar approaches leads to gene editing without double-stranded DNA breaks [64–67, and https://www.biorxiv.org/content/10.1101/2022.06.01.494256v1].

Besides limitations due to possible genotoxicity and applications of the protocols in clinical settings, all these studies concurrently demonstrated the possibility to use these highly efficient gene-editing protocols for personalized treatment and precision medicine of β-thalassemia.

## 4 CRISPR-Cas9 protocols for decreasing the excess of free α-globin

The pathophysiology of β-Thalassemia is strongly associated with an excess of free α-globin chains caused by the imbalance between α- and β-globin chains, especially in the case of β^0^-Thalassemia; this is the major factor leading to ineffective erythropoiesis and hemolysis (Schrier, 2002; Xie et al., 2007; Voon et al., 2008; Mettananda et al., 2015; Mettananda et al., 2017a). The reduction of free α-globin chains has a clear clinically beneficial impact, as suggested by studies demonstrating that when α-thalassemia is co-inherited with β-thalassemia, the excess free α-globin chains is in most cases significantly reduced, ameliorating the clinical severity. Furthermore, Lechauve et al. (2019) experimentally validated this hypothesis, demonstrating the role of the autophagy-activating kinase ULK-1 (Unc-51-like kinase 1) in promoting autophagy-associated controlled clearance of the free α-globin chains. Using β-thalassemic mice, it was found that the loss of the *ULK1* gene reduced autophagy, exacerbating the disease phenotypes, in association with a lack in the clearance of α-globin in red blood cell precursors. On the contrary, rapamycin-mediated ULK1 and autophagy reduce free α-globin accumulation in erythroid cells.

The expression of the α-globin genes was reduced by Mettananda et al. (2017b) using CRISPR/Cas9 genome editing to mimic a natural mutation causing α-thalassemia in association with a deletion of the MCS-R2 α-globin enhancer (Mettananda et al., 2017b). This CRISPR-Cas9 based approach might be of clinical relevance, as this strategy caused a reduction in α-globin expression and a correction of the globin chain imbalance, in erythroid-differentiated, gene-edited edited CD34^+^ cells from β-thalassemia patients.

A second study concerning this issue was published by Pavani et al. (2021), who were able to demonstrate a correction of the pathological phenotype of β-thalassemia by CRISPR/Cas9 editing of the α-globin locus in human hematopoietic stem cells. In this study, α-globin was downregulated, by *HBA2* gene deletion, in order to generate an α-thalassemia trait, with associated correction of α/β globin imbalance (Pavani et al., 2021).

## 5 Combined protocols based on gene therapy and gene editing

The translation of the gene editing procedures from pre-clinical studies to clinical therapeutic application has been sustained by the excellent results obtained by clinical trials based on the use of therapeutic lentivirus (LV) carrying a normal β-globin gene (Boulad et al., 2022; Locatelli et al., 2022; Magrin et al., 2022; Tucci et al., 2022; Segura et al., 2023). For instance, Locatelli and collaborators reported a Phase 3 clinical study (HGB-207, NCT02906202) carried on 23 patients receiving “betibeglogene autotemcel or beti-cel” treatment with a median follow-up of 29.5 months. This clinical trial was conducted on adult and pediatric patients with transfusion-dependent β-thalassemia and a non-β^0^/β^0^ genotype (Locatelli et al., 2022). The efficacy of the treatment was demonstrated by the finding that transfusion independence occurred in 20 of 22 patients, with an average hemoglobin level of 11.7 g per deciliter (range, 9.5–12.8) (Locatelli et al., 2022). Similar promising results were obtained by Magrin et al. (2022), reporting the data originating by the HGB-205 (NCT02151526) clinical trial.

An interesting development of LV vectors proposed for gene therapy of β-thalassemia is the linkage of the therapeutic β-globin gene to other useful gene sequences. An example of such bifunctional vectors has been described by Brusson et al., using as experimental model system hematopoietic stem and progenitor cells (HSPCs) isolated from sickle-cells disease (SCD) patients (Brusson et al., 2023). In this case, the employed bifunctional LV vector was expressing an anti-sickling β-globin (βAS3-globin) and an artificial microRNA specifically downregulating βS-globin expression (Brusson et al., 2023). The aim of this approach was to obtain a miRNA-mediated reduction of the HbS levels, favoring at the same time the incorporation of βAS3 into Hb tetramers. This study is an excellent proof-of-principle that clinically relevant end-points can be reached by bifunctional LV vectors combining gene addition and gene silencing strategies; furthermore, this study sustain the concept of combined treatments for β-thalassemia.

In this respect, the combination of gene therapy and gene editing was studied by Ramadier et al., who described two therapeutic approaches combining LV-based gene addition therapy ansd CRISPR-Cas9 gene editing (Ramadier et al., 2022). In both cases, the protocols were based on base-editing knock down the sickle β-globin in association with *de novo* expression of anti-sickling globin (AS3) or anti-sickling fetal γ-globins. While in this study expression of anti-sickling fetal γ-globins was obtained by gene addition (Ramadier et al., 2022), this can be also obtained by gene editing procedures for induction of γ-globin gene expression, as already described in Figure 2 and recently reported in the study by Venkatesan et al. 2023b). These studies confirmed also the possible use of combined protcols based on complementary gene editing procedures.

## 6 Multiplex CRISPR-Cas9 protocols

Han et al. (2022) recently published an interesting report demonstrating that CRISPR-Cas9-based gene editing approaches can be combined. This was also found by Psatha et al. (2021), who described a very interesting multiplex gene editing protocol based on the combination of two single gene editing strategies, one aimed at silencing the BCL11A repressor, the other aimed at disrupting the BCL11A binding sites of the γ-globin gene promoter (Psatha et al., 2021). The results obtained demonstrated that this CRISPR-Cas9 based multiplex genomic editing efficiently induced fetal hemoglobin expression.

## 7 Combining *de novo* production of HbA with HbF induction

In order to obtain increased levels of HbF together with *de novo* production of HbA, the possibility to perform CRISPR-Cas9 based multiplex genomic editing for BCL11A silencing (Frangoul et al.; Bjurström et al., 2016) and for correction of the primary mutation (Liang et al., 2017) might be considered. The advantage of this strategy is that both protocols use the same target cells (CD34^+^ cells) and, with respect to the expected therapeutic protocol, the same clinical steps for collecting the CD34^+^ cells to be gene-edited and for preparing the patients for the infusion of gene-edited cells (Bjurström et al., 2016; Fu et al., 2022). On the other hand, major drawbacks are expected, i.e., higher off-targeting effects and genotoxicity (Bothmer et al., 2017; Samuelson et al., 2021).

In this respect, while Han et al. reported no major increase of off-target effects of multiplex genomic editing (Han et al., 2022), on the contrary, Samuelson et al. have recently found that multiplex CRISPR-Cas9 genome editing in hematopoietic stem cells for HbF reinduction generates chromosomal translocations (Samuelson et al., 2021). This is not unexpected, since genomic instability is one of the major and still unresolved concerns in using gene editing strategies, including CRISPR-Cas9 based approaches (Bothmer et al., 2017). Recent reports described that gene editing might be associated to chromosomal aberrations with formation of micronuclei and chromosome bridges leading to copy number variation, telomeric portion loss, and chromotripsis (Cullot et al., 2019; Blattner et al., 2020; Leibowitz et al., 2021). Moreover, it should be considered that the identification of these alterations is not a simple task. In this respect, Holgersen et al. reported data strongly suggesting that *in silico methods* are of limited use for predicting the off-target effects of oligonucleotides, and RNA-seq based experimental screening should be instead considered the preferred approach (Holgersen et al., 2021). This raises further concerns in the transfer of gene editing technology from laboratory investigations to clinical settings.

In summary, the issue of genotoxicity of multiplex CRISPR/Cas9 based gene editing should be considered still an open issue, and caution should be suggested in using these multiplexed approaches. For these reasons, for HbF induction in combination with gene-editing approaches, it has been suggested to employ pharmacological HbF induction, using as first-choice repositioned drugs, or drugs already validated and used in clinical trials.

## 8 Induction of fetal hemoglobin: use of small-molecular weight molecules

The search for fetal hemoglobin inducers is a fast-moving field aiming to bring this approach from the laboratory to clinical settings. Review articles on HbF inducers are available discussing updates and the most recent findings (Gambari and Fibach, 2007; Sripichai and Fucharoen, 2016b; Lohani et al., 2018; Langer and Esrick, 2021; Bou-Fakhredin et al., 2022; Hashemi and Ebrahimzadeh, 2022; Prosdocimi et al., 2022). For preliminary screening of fetal hemoglobin inducers several *in vitro* cellular systems have been employed, such as In this case, several cellular model systems for the screening of HbF inducers are available, such as the K562 erythroleukemia cell line (Gambari and Fibach, 2007) or the HUDEP-1 cell line derived from umbilical cord blood cells (Papasavva et al., 2021). Of course, the effects of the identified HbF inducers should be confirmed and further characterized using primary cells isolated from β-thalassemia patients, such as erythroid precursor/progenitor cells (Gambari and Fibach, 2007). Table 3 reports a partial list of HbF inducers (Fucharoen et al., 1996; Marianna et al., 2001; Fibach et al., 2003; Lampronti et al., 2003; Fibach et al., 2006; Rönndahl et al., 2006; Aerbajinai et al., 2007; Zuccato et al., 2007; Moutouh-de Parseval et al., 2008; Fibach et al., 2012; Lulli et al., 2013; Shi et al., 2013; Reid et al., 2014; Bianchi et al., 2015; Gasparello et al., 2017; Kalantri et al., 2018; Iftikhar et al., 2019; Mettananda et al., 2019; Gholampour et al., 2020; Guo et al., 2020; Cheng et al., 2021; Zuccato et al., 2021; Iftikhar et al., 2022; Li et al., 2022; Zuccato et al., 2022; Takase et al., 2023), some of them presently considered in clinical trials (such as hydroxyurea and sirolimus). Remarkably, the proposed mechanisms of action (and the molecular/biochemical targets) are highly heterogenous. For instance, HbF inducers can inhibit DNA methylation, inhibit histone lysine methyltransferases, inhibit HDAC activity, activate the p38 MAPK pathway, inhibit the mTOR pathway, inhibit the binding of transcription factors (for instance Sp1, BCL11A) to the target DNA, inhibit the expression of γ-globin gene repressors (such as BCL11A and KLF6). Interestingly, several HbF inducers (for instance thalidomide, rapamycin, hydroxyurea) are repurposed/repositioned drugs, thus strongly facilitating the transfer of laboratory investigations to the clinical practice. Examples of clinical trials based on the HbF inducers enlisted in Table 3 are hydroxyurea (clinical trials NCT00001958 and NCT03183375), sirolimus (clinical trials NCT03877809 and NCT04247750), thalidomide (NCT05132270) and 2,2-dimethylbutyrate (HQK-1001) (NCT00790127).

**TABLE 3:** Fetal hemoglobin inducers.

Agent	Comments	References
5-Azacytidine	DNA hypomethylation	Kalantri et al. (2018)
Hydroxyurea	DNA hypomethylation	Fucharoen et al. (1996)
Butyrate	HDAC activity inhibition	Bianchi et al. (2015)
2,2-dimethylbutyrate (HQK-1001)	A phase II study of the efficacy and safety of 2,2-dimethylbutyrate (HQK-1001), an oral fetal globin inducer	Reid et al. (2014)
Valproate	Activation of p38 MAPK pathway, HDAC inhibition	Rönndahl et al. (2006)
Thalidomide derivatives	Repurposed drugs; Activation of p38 MAPK pathway	Aerbajinai et al. (2007); Moutouh-de Parseval et al. (2008); (Prosdocimi et al. (2022)
Trichostatin A	HDAC inhibition	Marianna et al. (2001)
Oridonin	Activation of p38 MAPK signaling	Guo et al. (2020)
Rapamycin	mTOR inhibitor	Fibach et al. (2006); Zuccato et al. (2022)
Everolimus	mTOR inhibitor	Zuccato et al. (2007)
Mithramycin	Inhibition of Sp1/DNA interactions	Fibach et al. (2003)
Resveratrol	Activation of p38 MAPK signaling	Fibach et al. (2012)
Angelicin	Induction of γ-globin expression via NRF2/ARE stress response pathway	Lampronti et al. (2003)
Cinchonidine, Quinidine and Cinchonine	*Cinchona* alkaloids potent inducers of the expression of γ-globin genes in erythroid cells	Iftikhar et al. (2019); Zuccato et al. (2021)
Tranylcypromine	Lysine-specific demethylase 1 (LSD1) inhibition	
Vorinostat	HDAC inhibition, reduces expression of α-globin, induces γ-globin expression	Mettananda et al. (2019)
Vasicinol and Vasicine	Induction of γ-Globin Genes in a Pre-Clinical Study of HbF Inducers isolated from *Adhatoda vasica*	Iftikhar et al. (2022)
miR-92a-3p	Inhibition of BCL11A	Li et al. (2022)
miR-210-3p	Inhibition of BCL11A	Gasparello et al. (2017)
miR-2355-5p	Upregulation of γ-globin gene expression by inhibiting KLF6	Cheng et al. (2021)
miR-30a	Regulation of γ-globin gene expression through targeting BCL11A	Gholampour et al. (2020)
miR-486-3p	Regulation of γ-globin gene expression through direct modulation of BCL11A	Lulli et al. (2013)
RK-701	Inhibitor of histone lysine methyltransferases G9a and G9a-like protein (GLP), that catalyze the dimethylation of histone H3 lysine 9 (H3K9me2), which acts as an epigenetic repressive mark	Takase et al. (2023)

## 9 Combining CRISPR-Cas9 based correction of the β-thalassemia mutation with the treatment of erythroid cells with HbF inducers

The idea of a combined approach for developing a therapeutic protocol for β-thalassemia based on gene therapy-mediated adult hemoglobin (HbA) production and fetal hemoglobin (HbF) induction is not new (Breda et al., 2013; Finotti et al., 2015). This is an important point since gene therapy might fail in reaching the complete reversion of the β-thalassemic phenotype (Breda et al., 2013; Finotti et al., 2015). In fact, following gene transfer, all or a large proportion of erythroid cells might express suboptimal levels of β-globin, significantly lowering the therapeutic potential of the gene therapy approach. These limitations might also be present in CRISPR-Cas9 based approaches aimed to reconstitute adult hemoglobin (HbA) production following gene editing (Cosenza et al., 2021). In this research field, Zuccato et al. were able to demonstrate the usefulness of the combination between gene therapy and HbF induction (Zuccato et al., 2012). Their end-point was the reduction in the treated ErPCs of the excess of free α-globin aggregates. Zuccato et al. demonstrated that gene therapy, performed with the lentiviral β-globin vector T9W, and HbF induction with mithramycin could be combined (Zuccato et al., 2012). In fact, the combination of T9W β-globin gene transfer together with fetal hemoglobin induction was far more efficacious than single treatments in removing the excess of α-globin proteins in β-thalassemic erythroid cells. This strategy has been reviewed by Finotti et al. (2015) and Breda et al. (2013). As observed in gene-therapy of β-thalassemia (Breda et al., 2013; Dong et al., 2013; Finotti et al., 2015; Karponi and Zogas, 2019; Thuret et al., 2022), the efficiency and duration of the correction of HbA production might fall below therapeutic levels also in gene editing (Cosenza et al., 2021); in this case, combined treatment with HbF inducers might be considered a useful fallback strategy to reach durable therapeutic hemoglobin levels in treated cells, with contribution of an hemoglobin (HbF) firmly established to be beneficial for β-thalassemia patients (Forget, 1998; Gambari and Fibach, 2007; Musallam et al., 2012; Sripichai and Fucharoen, 2016a).

**FIGURE 3:**
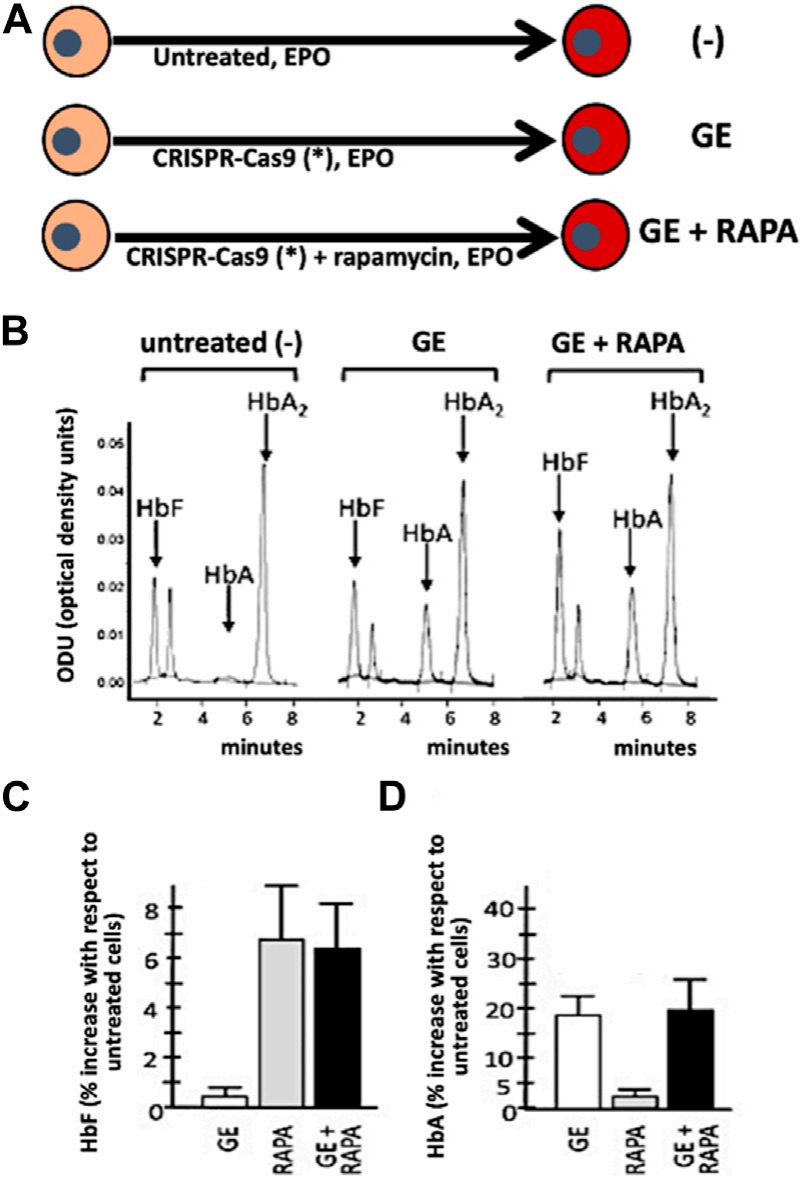
Co-treatment of ErPCs from β-thalassemia patients with the CRISPR-Cas9 based approach to correct the β^0^39-globin gene mutation (GE) and fetal hemoglobin induction following treatment with rapamycin (RAPA). **(A)** Pictorial representation of the experimental protocols. **(B–D)** Demonstration that in (GE + RAPA) treated cells both HbF **(B,C)** and HbA **(B,D)** are increased. Remarkably, in (GE + RAPA) treated cells HbF increase is similar to RAPA treated cells **(C)** and HbA increase is similar to GE-treated cells. **(B–D)** of this Figure are modified from Cosenza et al., with permission (copyright can be found at https://www.mdpi.com/2073-4425/13/10/1727) (Cosenza et al., 2022).

The first example of combined treatment using HbF inducers and gene editing was recently reported by Cosenza et al. (2022), who previously developed a protocol for CRISPR-Cas9-based gene correction of the β^0^39-thalassemia mutation, one of the most frequent in the Mediterranean area (Origa, 2017). The study was aimed at determining whether pharmacologic induction of HbF could be combined with *de novo* production of HbA obtained by the correction of the β^0^-thalassemia mutation using the developed CRISPR-Cas9 protocol (Cosenza et al., 2022) (Figure 3). As an inducer of HbF rapamycin (also known as sirolimus) (Sehgal, 2003), was selected. This lipophilic macrolide has been reported to be a strong inducer of HbF *in vitro* (Mischiati et al., 2004; Fibach et al., 2006; Zuccato et al., 2007; Pecoraro et al., 2015; Zuccato et al., 2022), *in vivo* animal model systems (Zhang et al., 2014; Khaibullina et al., 2015; Wang et al., 2016), in some patients affected by sickle-cell disease (SCD) (Gaudre et al., 2017; Al-Khatti and Alkhunaizi, 2019), and in a cohort of β-thalassemia patient participating to the NCT03877809 clinical trial (A Personalized Medicine Approach for β-thalassemia Transfusion Dependent Patients: Testing sirolimus in a First Pilot Clinical Trial) (Zuccato et al., 2022). The data obtained by Cosenza et al. support the concept that CRISPR-Cas9 mediated gene editing restores HbA production in erythroid precursor cells from homozygous β^0^39/β^0^39 homozygous patients, together with pharmacological induction of HbF. Remarkably, no decrease of HbF in gene edited cells occurs (Cosenza et al., 2021; Cosenza et al., 2022) and no evidence of a decrease of HbA following HbF induction in gene edited cells has been reported (Cosenza et al., 2022), suggesting that this might be considered an interesting protocol to minimize side effects (single plex CRISPR-Cas9 intervention is performed) together with maximization of total hemoglobin production (*de novo* production of HbA and increased production of HbF).

Furthermore, it is expected that the pharmacologic induction of HbF can be employed in co-treatment protocols together with gene editing procedures correcting other β-thalassemia mutations. These pre-clinical studies might be facilitated by the availability of β-thalassemia cellular bio banks, avoiding the need of new recruitment of β-thalassemia patients (Cosenza et al., 2016).

## 10 Conclusion and future perspectives

In our opinion, the possible combination between CRISPR-Cas9 gene editing and HbF induction deserves further studies to determine the real impact in real life for the management of β-thalassemia patients. While it is likely that the use of gene editing will be considered with high caution, due to the possible genotoxic effects of this strategy (Bothmer et al., 2017; Cullot et al., 2019; Blattner et al., 2020; Leibowitz et al., 2021; Samuelson et al., 2021), we would like to remind that clinical trials based on the use of CRISPR-Cas9 are ongoing. Examples are β-thalassemia and SCD patients infused with CRISPR-Cas9 modified autologous CD34^+^ HSPCs (NCT03655678; NCT03745287). Encouraging results were obtained, including the fact that a) some transfusion-dependent β-thalassemia patients became transfusion-independent already from the first months after infusion and b) one SCD patient was free of vaso-occlusive crises after treatment (Frangoul et al., 2021). With respect to possible combined co-treatment with HbF inducers, it should be underlined that both gene editing and HbF induction are expected to be carried on at sub-optimal conditions, trying to avoid unwanted side effects.

In addition, we have to mention that novel gene-editing strategies (for instance base-priming and base-editing), characterized by lower level of genotoxicity, are available and are expected to be extensively studied and validated in the next future (Liang et al., 2017; Zipkin, 2019; Collantes et al., 2021; Zhang et al., 2022; Badat et al., 2023; Hardouin et al., 2023). In our opinion, this last development of gene editing protocols is of great interest to propose combined treatments based on gene editing (or multiplexed gene editing) with HbF inducers.

A limit of the review is that it is based on few key papers reporting gene-therapy and CRISPR-CAS9-based gene-editing in combination with the use of HbF inducers (Zuccato et al., 2012; Breda et al., 2013; Cosenza et al., 2022). Our review might stimulate further research on this field, in order to verify the real impact of this approach.

In these studies, the choice of the HbF inducer is critical. We suggest employing repurposed drugs that a) increase HbF in cultures from β-thalassemia patients with different basal HbF levels; b) increase the overall Hb content per cell; c) selectively induce γ-globin mRNA accumulation, with only minor effects on β-globin and α-globin mRNAs; d) are currently under investigations in clinical trials, such as hydroxyurea (FDA approved for sickle-cell disease) (Yasara et al., 2020; Yasara et al., 2022), sirolimus (Zuccato et al., 2022), thalidomide (Chen et al., 2021) and 2,2-dimethylbutyrate (HQK-1001) (Reid et al., 2014).

The interest in combined therapies based on the use of gene editing and HbF induction is also due to the fact that HbF inducers might exhibit mechanism(s) of action enforcing erythroid cells to be activate biological processes of great importance for clinical treatments. For instance, in the case of β-thalassemia, a key end point is represented by the reduction of the excess of α-globin chains, as reported in several studies (Schrier, 2002; Xie et al., 2007; Voon et al., 2008; Mettananda et al., 2015; Mettananda et al., 2017a). In this respect, rapamycin has been shown to induce with high efficiency (in addition to increased HbF production) autophagy, thereby decreasing the excess of free α-globin chains (Lechauve et al., 2019; Buffa et al., 2023; Zurlo et al., 2023). In our opinion this double effect of rapamycin, might be used in pre-clinical studies also in combination with gene editing strategies to maximize the decrease expression of α-globin genes in treated cells.
